# Croatian success in early breast cancer detection: favorable news in Breast Cancer Awareness Month

**DOI:** 10.3325/cmj.2020.61.389

**Published:** 2020-10

**Authors:** Boris Brkljačić, Andrea Šupe Parun

**Affiliations:** 1 Department of Diagnostic and Interventional Radiology, University Hospital Dubrava, University of Zagreb School of Medicine, Zagreb, Croatia; 2 Croatian Institute of Public Health, Zagreb, Croatia

Breast cancer is by far the most common cancer in women worldwide, both in developed and developing countries. Each year, around two million people are diagnosed with the disease and 600 000 die from it (1). Due to an increase in life expectancy, in most countries breast cancer incidence has been steadily rising.

The Joint Research Centre (JRC) of the European Commission has just released new estimates of cancer burden in each of the European Union (EU-27) countries for 2020. The data were obtained from population-based cancer registries that are a part of the European Network of Cancer Registries and World Health Organization mortality database. According to these estimates, breast cancer is the most commonly diagnosed malignant disease and the most common malignant cause of death in the EU (16.5%), followed by lung (15.6%) and colorectal cancer (12.4%) (2).

Age-standardized incidence rates for all cancers position Croatia near the EU-27 average (15th out of 27 countries; 14th in men and 19th in women), but cancer mortality rates place it fifth in Europe, preceded only by Slovakia, Poland, Cyprus, and Hungary. When we look only at breast cancer estimates for Croatia, the situation is much better. Standardized breast cancer mortality rates for Croatia are below the EU-27 average (16th out of 27 countries) (2).

According to the last available data for Croatia, in 2017 breast cancer was diagnosed in 2767 patients (rate 132.1/100,000), and in 2019, 752 women died from the disease (rate 35.9/100,000). Breast cancer is the third most common malignant cause of death in women in Croatia, preceded by lung and colorectal cancer. However, the number of women dying from breast cancer has been steadily decreasing for four years in a row (3,4).

Although the majority of breast cancer cases are not preventable, it is crucial to make the diagnosis at an early stage, when more than 90% of women can be cured. Mammography screening remains the best and widely validated method of early detection of breast cancer, despite its shortcomings and the emergence of other imaging modalities with high diagnostic accuracy.

Mammography screening for breast cancer has a long history in European countries. Austria introduced first opportunistic screening program in 1974, while Finland was the first country to introduce a population-based screening for breast cancer in 1987. Only two years later, the program reached national coverage. Although in the majority of other European countries, national coverage was achieved only in the 2000s, the program has since been successful in reducing breast cancer mortality (5). European Society of Breast Imaging and 30 national breast radiology bodies from European countries support mammography for population-based screening and recommend the involvement of radiologists qualified as screening readers and the use of double reading (6).

In Croatia, national program for early breast cancer detection was implemented in 2006, with significant involvement by expert radiologists, epidemiologists, other clinicians, and public health experts. As part of the program, all women aged 50-69, are invited to attend a mammogram appointment every two years. The goal is to reduce breast cancer mortality, detect cancer in early stage, and improve the quality of life of women diagnosed with breast cancer (7). The program is currently in its sixth cycle, with around 150 000 mammograms performed every year. In the first four cycles, 5422 breast cancers were detected, and about 7000 breast cancers have been detected so far. We can conclude that the program showed promising results in the early detection rate and the reduction of the number of patients with regional and distant metastasis (8,9).

Early diagnosis improves cancer outcomes by enabling care provision at the earliest possible stage and is therefore an important public health strategy in all settings. Professional radiology societies encourage creating dedicated pathways for high-risk women to be offered breast MRI according to national or international guidelines and recommendations (6).

Due to the synergy of experts and increasing response of target population, the national program for early breast cancer detection in Croatia has yielded promising results. As October has been proclaimed the Breast Cancer Awareness Month, we are especially proud to present the promising results of our program in the October issue of the *Croatian Medical Journal*.
